# Survival strategy of the endangered tree *Acer catalpifolium* Rehd., based on ^13^C fractionation

**DOI:** 10.1002/ece3.6600

**Published:** 2020-08-05

**Authors:** Wenchen Song, Yanhong Liu

**Affiliations:** ^1^ College of Forestry Beijing Forestry University Beijing China; ^2^ College of Life and Environment Sciences Minzu University of China Beijing China

**Keywords:** ^13^C, *Acer catalpifolium*, endangered plant, plant protection, survival strategy

## Abstract

We conducted a field investigation and evaluation of ^13^C natural abundance to determine the growth habit and propagation strategy of *Acer catalpifolium* Rehd., a tree species native to China that is highly endangered. The results showed that *A*.* catalpifolium* is a K‐selected strategist and pioneer species. Its narrow ecological range limits its geographical distribution, and poor fecundity limits its population size. The analysis of ^13^C natural abundance showed that *A*.* catalpifolium* does not use organic matter for reproduction when its stand volume is less than 1.08 × 10^6^ cm^3^ or it is less than 18.6 m tall, but it does use this strategy when it has a sufficient 1.08 × 10^6^ cm^3^ stand volume or more or is taller than 18.6 m. If environmental conditions are not conducive (e.g., severe human disturbance, cliff edges, or fierce interspecific competition) to the continued growth of the tree, *A*.* catalpifolium* may allocate organic matter for reproduction. Human disturbance seems to promote the population expansion of *A*.* catalpifolium*. We provide our suggestions for the promotion and protection of *A*.* catalpifolium* as a species.

## Graphical abstract

This study involved a field investigation and evaluation of ^13^C natural abundance to determine the growth habit and propagation strategy of Acer catalpifolium Rehd., a tree species native to China that is highly endangered. Its narrow ecological range limits its geographical distribution, and poor fecundity limits its population size. The analysis of ^13^C natural abundance showed that *A*.* catalpifolium* allocates organic matter for reproduction only when it has accumulated sufficient biomass and height or when environmental conditions are not conducive to continued growth.

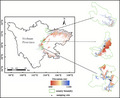

## 1 INTRODUCTION

*Acer catalpifolium* Rehd. is a tree species native to China that is highly endangered. It belongs to the family Aceraceae and is closely related to *Acer miaotaiense* (Wang, He, Xu, Peng, & Zhao, 2019). At one time, it was believed that only 53 trees remained, and the species was listed in the “wild plants with extremely small populations (WPESP) rescue and protection plan” (State Forestry Administration of China, 2010). The present survey revealed 206 trees, including 102 adult trees and 43 saplings, scattered in Sichuan Province. A previous study showed that there were more than 200 trees in Guizhou Province (Wu, Long, & Qing, 2018). Nevertheless, the species remains extremely endangered with a very small population. At present, the only studies of this species have used seedlings to evaluate the effects of water and light stress (Zhang et al., 2019), leaf nutrients (Gao, Song, & Liu, 2019), and population structure and dynamics (Wu et al., 2018; Xu & Liu, 2019). These studies do not provide enough information to support the rescue and protection of *A*.* catalpifolium*. To support a species that persists in small and isolated populations, the best approach is to restore genetic diversity and adaptive potential through natural interventions (Stowell, Pinzone, & Martin, 2017). The first step in doing so is to understand the survival strategy of *A*.* catalpifolium*.

Natural ^13^C methods have been widely used to investigate the processes occurring at the plant–environment interface (Gautam & Lee, 2016). δ^13^C values have often been used to calculate the intrinsic water use efficiency (iWUE) using the Carbon Isotope Photosynthesis Model of Plants created by Farquhar, Ehleringer, and Hubick (1989). With the development of plant isotope physiology (especially tree‐ring analysis), more studies have focused on the ^13^C fractionation between leaves and stems (Δ_L‐S_). Richardson et al. (2015) stated that carbon cycle models should include a “two‐pool model structure,” in which tree growth could access a “young pool” of organic matter produced previously and an “old pool” of organic matter with much older carbohydrates. The two‐pool model was strongly supported by data on carbon isotope ratios (McCarroll, Whitney, Young, Loader, & Gagen, 2017). In general, leaves are ^13^C‐depleted compared with all other plant organs (e.g., roots, stems, fruits) (Ghashghaie & Badeck, 2014), and the δ^13^C values of tree leaves are correlated with iWUE but those of other organs are not (Konate, Dreyer, & Epron, 2016). Therefore, Δ_L‐S_ could reflect the process of carbon partitioning in trees that results in the two‐pool situation (Gessler et al., 2014; McCarroll et al., 2017).

In the present study, a field investigation of growth habits and an evaluation of ^13^C natural abundance were conducted to determine the growth and propagation strategy of *A*.* catalpifolium*. Using the results, we provided suggestions for the rescue and protection of *A*.* catalpifolium*.

## 2 METHODS

### 2.1 Field investigation and sampling

We searched for scattered adult *A*.* catalpifolium* individuals in Sichuan Province and recorded their locations, diameter at breast height (DBH) measurements, heights, and environmental conditions. Samples of leaves and shoot tips (10 replicates) were obtained in July from the tops of newly growing branches of the trees. If there were samaras (fruits) on the plant, some of them were also collected as samples. Seeds were collected and planted in experimental plots (see Zhang et al., 2019 for more detail) to evaluate seed production, germination rate, and survival rate of intact seeds. Samples of leaves and branches of 100 adult *A*.* catalpifolium* specimens were collected, as well as 29 samples of samaras collected from fruitful trees. These samples were analyzed to obtain their levels of δ^13^C, C, N, and P, using a Finnigan MAT 253 Isotope Ratio Mass Spectrometer (Flash 2000 EA‐HT Elemental Analyzer; Thermo Fisher Scientific Inc., USA).

**Figure 1:**
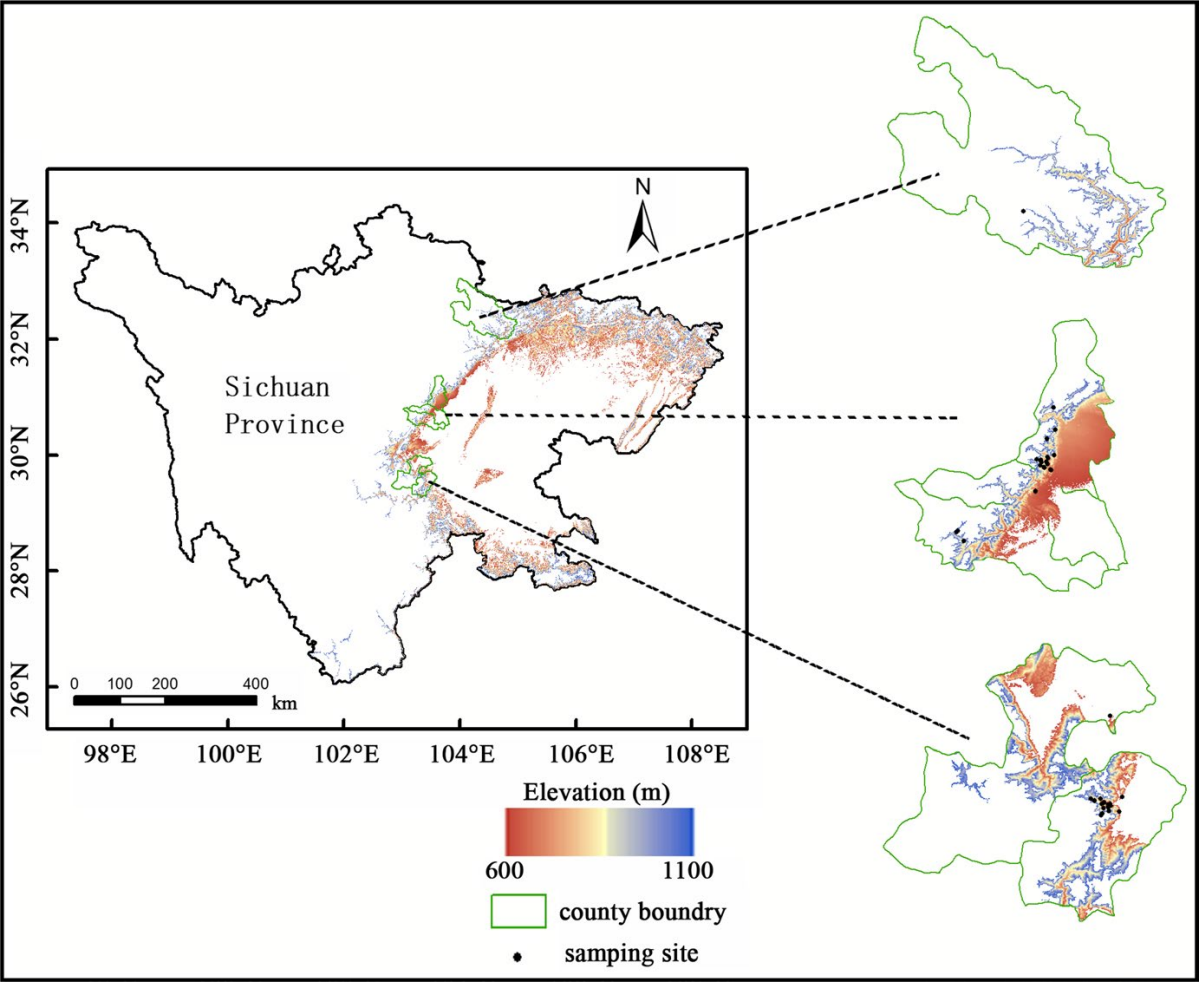
The distribution of *Acer catalpifolium* Rehd. in Sichuan Province

The trees were distributed in a narrow arc in a mountainous area in Sichuan Province (97°21′—108°33′E, 26°03′—34°19′N) between 600 and 1,100 meters in elevation (Figure 1). They were very widely dispersed, with only one or two plants in many plots, and were found growing on almost every kind of land in the distribution area, including some poor conditions such as on cliffs, in stone crevices, and cracks in cement. The deciduous period of *A*.* catalpifolium* is very short, less than a month, and for some individuals as short as a few days. However, the fruit‐bearing period of *A*.* catalpifolium* is very long, and the samaras hang on trees almost all year round. The reproductive capacity of *A*.* catalpifolium* is very poor. The fruitful trees composed less than 30% of the total, and the rate of seeds in samaras was less than 5%. Most fruitful trees were found in human‐disturbed environments (Table S1). Although the rate of seed production was low, the germination rate and survival rate of intact seeds were both very high, more than 90%.

### 2.2 Stable isotope analysis

The ^13^C photosynthetic discrimination (Δ), which is correlated with iWUE, was calculated using the model described by Farquhar et al. (1989):

(1) $$\Delta =\frac{\left(\delta^{13}C_{L}‐\delta^{13}C_{A}\right)}{\left(\frac{\delta^{13}C_{A}}{1000}+1\right)}\times 1000‱,$$

where δ^13^C_A_ and δ^13^C_L_ represent δ^13^C for atmospheric CO_2_ and tree leaves, respectively.

The δ^13^C fractionation between the leaves and stems of trees (Δ_L‐S_), which was correlated with the changes in ^13^C abundance of leaves and stem after a series of processes, was calculated as follows:

(2) $$\Delta_{L‐S}=\frac{\left(\delta^{13}C_{L}‐\delta^{13}C_{S}\right)}{\left(\frac{\delta^{13}C_{S}}{1000}+1\right)}\times 1000‱,$$

where δ^13^C_S_ represents δ^13^C for tree stems (Gessler et al., 2014).

Similarly, the δ^13^C fractionation between leaves and samaras of trees (Δ_L‐P_), which was correlated with the changes in ^13^C abundance of leaves and stems after a series of processes, was calculated as follows:

(3) $$\Delta_{L‐P}=\frac{\left(\delta^{13}C_{L}‐\delta^{13}C_{P}\right)}{\left(\frac{\delta^{13}C_{P}}{1000}+1\right)}\times 1000‱,$$

where δ^13^C_P_ represents δ^13^C for tree samaras.

### 2.3 Statistical analysis

The values presented in the figures are given as means ± standard errors of means. The distribution map was drawn by ArcGIS 10.2 (ESRI Inc., CA, USA). All data analyses were performed with IBM SPSS Statistics 23.0 (IBM Inc., NY, USA).

## 3 RESULTS

**Figure 2:**
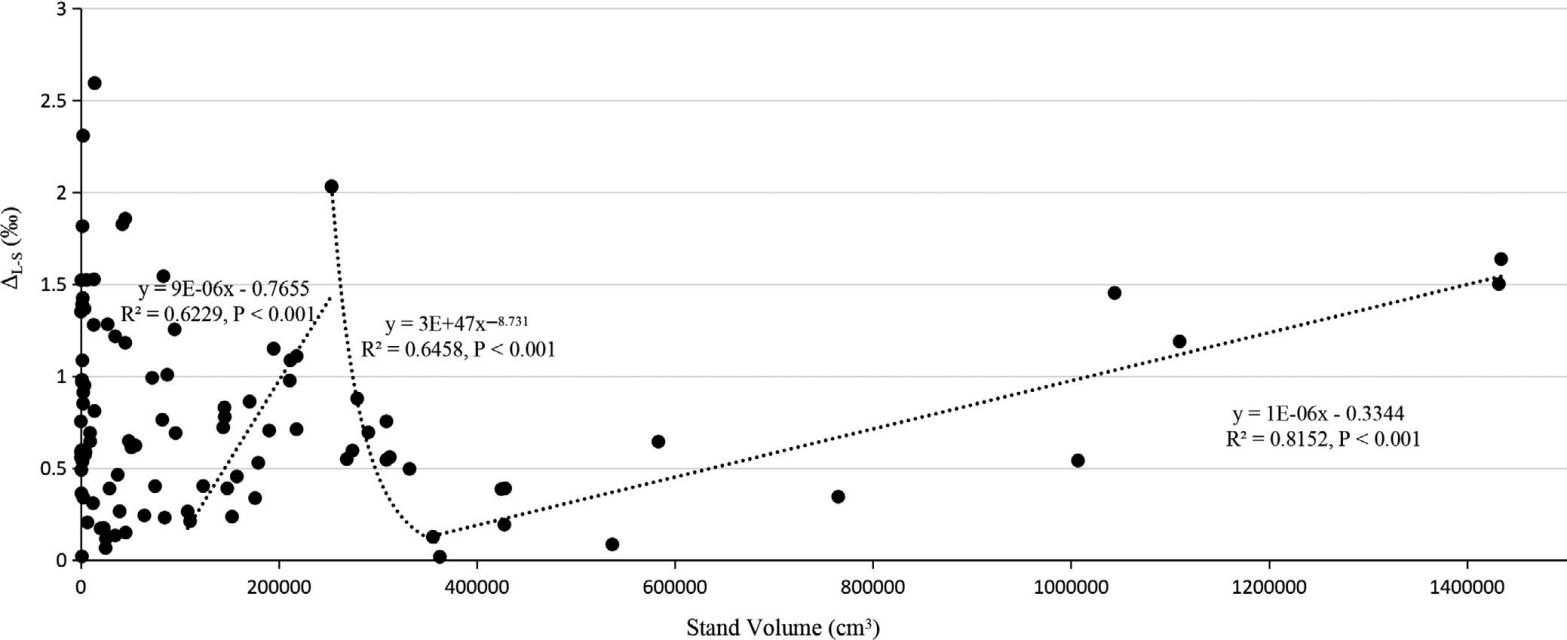
The correlation between the square of stand volume, the diameter at breast height (DBH) multiplied by tree height, and the δ^13^C fractionation between the leaves and stems of trees (Δ_L‐S_)

According to the turning point in the trend lines, Δ_L‐S_ changed continuously through the four stages with the increase in stand volume (the square of DBH was multiplied by tree height). When the stand volume was less than 1,079,707 cm^3^, Δ_L‐S_ was not significantly associated with the increase in stand volume; between 1,079,707 and 2,532,594 cm^3^, Δ_L‐S_ was significantly positively correlated with stand volume; between 2,532,594 and 3,625,851 cm^3^, Δ_L‐S_ was significantly negatively correlated with stand volume in power function; at values more than 3,625,851 cm^3^, Δ_L‐S_ was also significantly positively correlated with stand volume (Figure 2).

**Figure 3:**
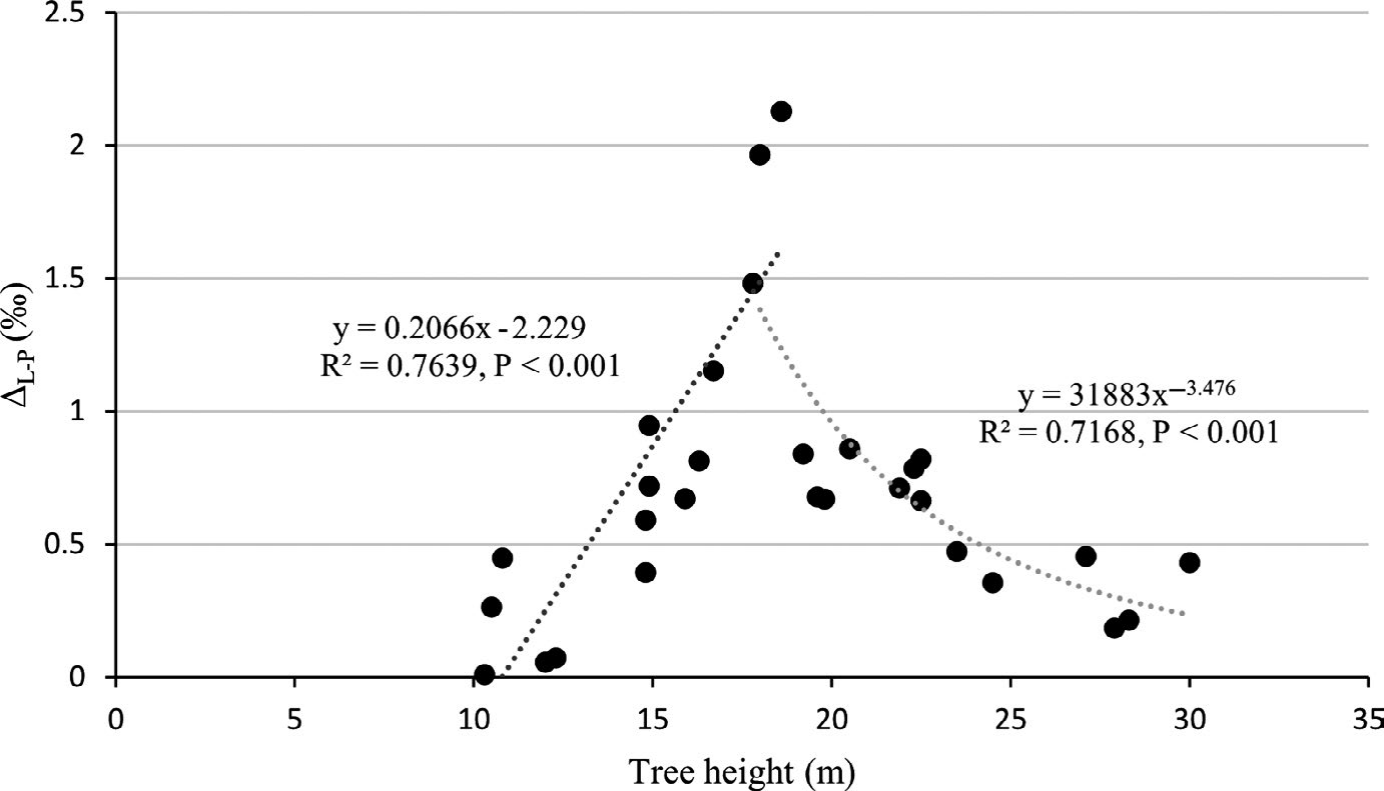
The correlation between the tree heights and the δ^13^C fractionation between leaves and samaras of trees (Δ_L‐P_)

**Figure 4:**
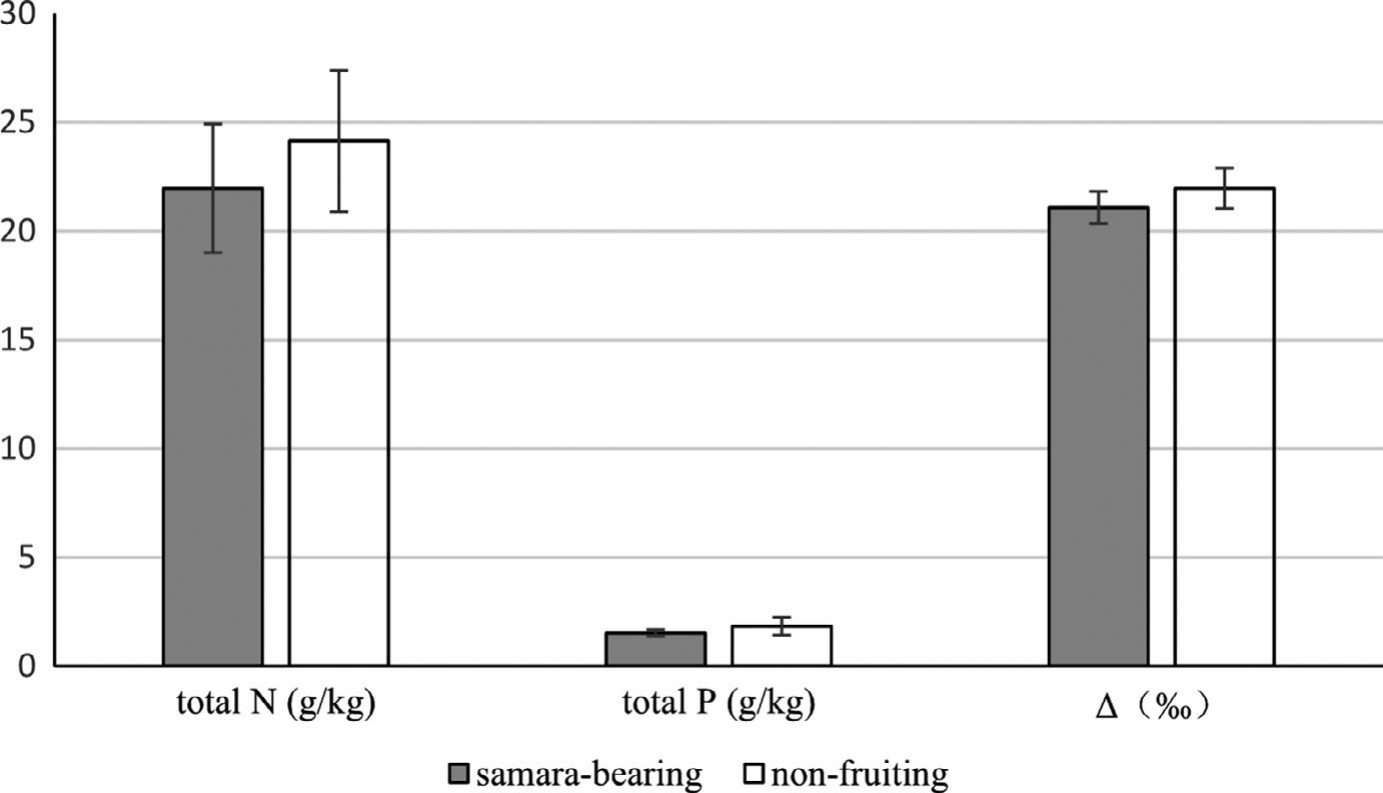
The amounts of N and P and the ^13^C photosynthetic discrimination (Δ) of leaves from samara‐bearing and non‐fruiting trees

Δ_L‐P_ changed continuously through two stages with the increase of tree height. When tree height was less than 18.6 m, Δ_L‐P_ was significantly positively correlated with tree height. When tree height was more than or equal to 18.6 m, Δ_L‐P_ was significantly negatively correlated with tree height in power function (Figure 3). The amounts of N and P and the ^13^C photosynthetic discrimination (Δ) of leaves on trees that bore fruit were lower than those of fruitless trees (Figure 4), but there was no significant difference in the amount of C between the two types of trees.

## 4 DISCUSSION

### 4.1 Growth habit and distribution

A previous report indicated that *A*.* catalpifolium* was found at elevations of 500–1300 m (State Forestry Administration of China, 2010). However, we found the trees only between 600 and 1,100 m of elevation, an even more narrow range than once believed. *A*.* catalpifolium* cannot adapt to cold, hot, or dry conditions or environments with strong solar radiation (Zhang et al., 2019), which limits them from spreading to the Chengdu Plain on the East, Qinghai‐Tibet Plateau on the West, or the cold, dry North. Only one arc corridor allows *A*.* catalpifolium* to spread to Guizhou Province, which is southeast of Sichuan Province. However, the corridor is in danger of being closed owing to the advance of global warming (Wischnewski et al., 2011). Such a geographical pattern greatly limits the distribution of *A*.* catalpifolium*.

A DNA sequencing study showed that *A*.* catalpifolium* is more closely related to *Acer* trees in the north of Sichuan Province (Wang et al., 2019). Compared with its deciduous broad‐leaved relatives from the North, *A*.* catalpifolium* seems to be evolving to adapt to the warmer environment in Sichuan. Characteristics like the very short defoliation period, a longer period of growth, and a longer reproduction period make *A*.* catalpifolium* more likely than other evergreen trees to thrive in a warmer habitat. Furthermore, unlike related plants that are r‐selected, the poor reproductive capacity of *A*.* catalpifolium*, as well as its much taller height, longer life span, higher germination rate and survival rate of intact seeds, strong interspecific competitiveness, and environmental adaptability show that it is a K‐selected strategist. However, the species still retains some characteristics of an r‐strategist, such as bearing many samaras, although most do not contain seeds. According to the population structure of *A*.* catalpifolium*, the age class of the species is not consistent and trees are blocked by regeneration obstacles, like other pioneer species in the community (Wu, Zheng, & Ma, 2002; Xu & Liu, 2019). This characteristic is also found in related species.

### 4.2 Growth and propagation strategy

When stand volume was less than 1,079,707 cm^3^, Δ_L‐S_ was not significantly associated with the increase in stand volume. This shows that *A*.* catalpifolium* is greatly affected by the environment at its sapling stage, and trees themselves are divided between using young carbon pools and old carbon pools. However, over time, *A*.* catalpifolium* shows an increasing accumulation of tree mass, gaining a certain amount of stored carbon. After reaching about 1.08 × 10^6^ cm^3^, trees are more and more inclined to use the stored old carbons, making the growth process more stable, to enhance their environmental adaptability and stress resistance. Therefore, when stand volume is between 1,079,707 and 2,532,594 cm^3^, Δ_L‐S_ was significantly positively correlated with stand volume. As trees continue to grow, their environmental adaptability and stress resistance increase, and their productivity begins to meet their needs for growth. As trees age, they are increasingly inclined to use the pool of newly synthesized young carbon. Therefore, when stand volume is between 2,532,594 and 3,625,851 cm^3^, Δ_L‐S_ was significantly negatively correlated with stand volume in power function. When stand volume exceeded 3,625,851 cm^3^, the productivity of trees could not continue to increase, and they began to gradually increase the proportion of old carbon they used. At this time, Δ_L‐S_ was also significantly positively correlated with stand volume.

When tree height was less than 18.6 m, Δ_L‐P_ was significantly positively correlated with tree height. When tree height was greater than or equal to 18.6 m, Δ_L‐P_ was significantly negatively correlated with tree height in power function. Thus, trees that were less than 18.6 m tall tended to use the old carbon pool for reproduction, whereas trees that were more than 18.6 m tall tended to use the young carbon pool for reproduction. Tree height is mainly affected by water supply, photosynthesis, nutrient supply, and xylem width, and the increase in tree height depends on these factors (Givnish, Wong, Stuart‐Williams, Holloway‐Phillips, & Farquhar, 2014). As their height increases, trees can get more light, increasing their ability to perform photosynthesis and grow. Trees that are less than 18.6 m tall show low productivity and competitiveness. *A*.* catalpifolium* is likely to choose growth over reproduction, and it is more inclined to use stored organic matter for propagation. Trees that are taller than 18.6 m show strong productivity and competitiveness, and are capable of both growth and reproduction. Therefore, the proportion of young organic carbon used for reproduction gradually rises.

The amounts of N and P and the Δ values of leaves on trees with fruit were lower than those of fruitless trees, which means that the samara‐bearing trees were less healthy than the fruitless trees. If the environment is not conducive to growth, the tree shifts its original growth‐oriented strategy to use some of the young organic carbon for reproduction rather than growth. This phenomenon explains why 27 of the 29 samara‐bearing trees were growing in an unfavorable environment, and why the shorter trees (less than 18.6 m tall) were more likely to use young carbon pools for propagation. The only two samara‐bearing trees growing in suitable places both had stand volume of more than 3,625,851 cm^3^ and were taller than 18.6 m, which indicates that these two trees are not affected by environmental factors. They were able to produce samaras because of their high productivity and had spare capacity to allocate part of their resources for reproduction, as well as growth. This explains why trees with a stand volume of greater than 3,625,851 cm^3^ gradually increase the proportion of old carbon pools that they use, causing a significantly positive correlation between Δ_L‐S_ and stand volume.

### 4.3 Influence of human disturbance

Of the 29 samara‐bearing trees, 24 were growing in harsh environments (e.g., severe human disturbance, cliff edges). Three of them grew in a state of fierce competition. Only two big trees with a stand volume of greater than 3,625,851 cm^3^ were growing in suitable places, and even they were only 4 m away from artificial roads. In a harsh environment with serious human disturbance, such as hardened ground, courtyard walls, and roads, plants have to contend with air pollution, a thin litter layer, and barriers to extending their roots. It is difficult for *A*.* catalpifolium* to obtain nutrients from the soil, which is why the N and P amounts and Δ in the leaves of samara‐bearing trees were significantly lower than those of non‐fruiting trees. In addition, a previous study showed that a too‐thin litter layer will induce N limitation on top of the existing P limitation, thus seriously affecting the growth of *A*.* catalpifolium* (Gao et al., 2019). Because disturbed environments are not conducive to the continued growth of trees, the trees may allocate a certain amount of organic matter for reproduction, to expand the distribution of the population, find more conducive habitats, and increase the chances that the population will survive. However, the bare land created by human disturbance also provides an opportunity for the *A*.* catalpifolium* seeds to sprout. This explains why the present study found more sapling trees in Sichuan Province and a previous study found more large trees in an area of Guizhou Province that is protected from human disturbance (Wu et al., 2018). In a sense, human disturbance promoted the population expansion of *A*.* catalpifolium*. However, according to our survey and visit, many local people are indifferent to environmental values and regard the seedlings as weeds to eradicate. Even the trees that grow are often cut down because they are “useless,” which seriously restricts the *A*.* catalpifolium* population.

## 5 CONCLUSION

*A*.* catalpifolium* is a K‐strategist and pioneer species. Its narrow ecological range limits its geographical distribution, and poor fecundity limits its population size. Whether the trees use photosynthates for growth or reproduction depends mainly on two factors. *A*.* catalpifolium* does not use organic matter for reproduction when its biomass is too small or it is not tall enough, but it does use organic matter for reproduction when it has a sufficient biomass and is tall enough. However, if environmental conditions are not conducive to the continued growth of the tree, *A*.* catalpifolium* may allocate organic matter for reproduction rather than growth. Human disturbance seems to promote the population expansion of *A*.* catalpifolium*.

To rescue and protect *A*.* catalpifolium*, we recommend basing management strategies on the growth stages of trees. Tree height and stand volume are important indicators of the tree growth stage. When tree biomass is small, we should create suitable habitats as far as possible and halt all kinds of habitat destruction. When trees grow tall and develop high biomass, we should increase the environmental pressure appropriately to stimulate the trees to use part of their organic matter for reproduction. Ex situ conservation is a good way to overcome geographical isolation and expand the distribution of *A*.* catalpifolium*. The bare land caused by human activities, especially on both sides of the road in valleys, can be used as a new habitat for *A*.* catalpifolium*. Such bare land also provides new corridors along which the species can spread. Last but not least, local governments should use propaganda and education to raise people's awareness of this endangered tree and prevent the small number of seedlings that do grow from being destroyed wantonly. Only in these ways can we adequately safeguard *A*.* catalpifolium*.

## CONFLICTS OF INTEREST

The authors declare no conflicts of interest.

## AUTHOR CONTRIBUTION

**Wenchen Song:** Conceptualization (lead); Data curation (lead); Formal analysis (lead); Investigation (lead); Methodology (lead); Validation (lead); Writing‐original draft (lead). **Yanhong Liu:** Funding acquisition (lead); Resources (lead).

### OPEN RESEARCH BADGES

This article has been awarded Open Data, Open Materials, Preregistered Research Designs Badges. All materials and data are publicly accessible via the Open Science Framework at https://doi.org/10.5061/dryad.44j0zpcbq; https://doi.org/10.5061/dryad.44j0zpcbq; https://doi.org/10.5061/dryad.44j0zpcbq.

## Supporting information

Table S1

## Data Availability

Data from this study can be seen in https://doi.org/10.5061/dryad.44j0zpcbq. Details of locations and environmental conditions are not suitable for publication, because someone may find these endangered trees and damage them, it would be detrimental to the conservation status of this endangered species to make them public.
