# Supplementary material for: Survival strategy of the endangered tree Acer catalpifolium Rehd., based on 13C fractionation
Source: Ecol Evol. 2020 Aug 5;10(16):8532–7. doi: 10.1002/ece3.6600 (PMC7452789; doi:10.1002/ece3.6600)
Supplement: Supplementary file 1 — Table S1 [file ECE3-10-8532-s001.docx]

Table S1. The diameter at breast height (DBH), tree height, the square of DBH multiplied by tree height (D^2^H), and surrounding environment of *Acer catalpifolium*.

| sample ID | diameter at breast (cm) | tree height (m) | stand volume (cm^3^) | surrounding environment of the tree |
| --- | --- | --- | --- | --- |
| QCS-26 | 9.5 | 10.3 | 92957.5 | On the stone platform, 3 m away from the road, there are many competing trees around it. (poor distribution habit) |
| DYZ-5 | 9.5 | 10.5 | 94762.5 | On a 70^o^ steep slope on a rocky cliff. (poor distribution habit) |
| QCS-14 | 11.3 | 10.8 | 137905.2 | Against the courtyard wall. (poor distribution habit) |
| DES-5 | 12.9 | 16.3 | 271248.3 | Near the highway, surrounded by five larger trees. (poor distribution habit) |
| YHQ-1 | 16.8 | 12.3 | 347155.2 | On a 60^o^ cliff, 3 m from the road (poor distribution habit) |
| ZGS-2 | 22.2 | 16.7 | 823042.8 | On the slope, there are many competing trees around it. (poor distribution habit) |
| TGS-1 | 21.5 | 20.5 | 947612.5 | On the edge of a cliff (poor distribution habit) |
| CCC-9 | 26.7 | 14.8 | 1055077 | On a 45^o^ cliff, 3 m from the road (poor distribution habit) |
| QCS-16 | 22.3 | 21.9 | 1089065 | On an artificial stone-cement slope (poor distribution habit) |
| QCS-7 | 24.9 | 17.8 | 1103618 | Against the highway. (poor distribution habit) |
| CCC-8 | 28.8 | 14.9 | 1235866 | In a bamboo forest of village. (poor distribution habit) |
| QCS-21 | 32.6 | 12 | 1275312 | On a stone slope, 3 m from the road. (poor distribution habit) |
| QCS-25 | 28.5 | 15.9 | 1291478 | In the hardened cement ground at the entrance of the cemetery. (poor distribution habit) |
| ZSQ-1 | 31.5 | 14.9 | 1478453 | On a 60^o^ steep rocky cliff. (poor distribution habit) |
| TGS-6 | 29.8 | 19.2 | 1705037 | On a 30^o^ slope, less than 1 m from another large tree. (poor distribution habit) |
| BRS-1 | 34.8 | 14.8 | 1792339 | Against the corner of temple wall. (poor distribution habit) |
| WNC-1 | 34.8 | 18 | 2179872 | On the ridge, next to the canal. (poor distribution habit) |
| SQ-3 | 38 | 18.6 | 2685840 | In a village. (poor distribution habit) |
| QCS-24 | 38.2 | 19.8 | 2889295 | Close to right-angle cement platform. (poor distribution habit) |
| TGS-5 | 37.2 | 22.3 | 3085963 | On a 30^o^ slope, surrounding hardened ground, 4 m away from the road. (poor distribution habit) |
| FHS-13 | 39.9 | 19.6 | 3120340 | In the hardened cement ground of the parking lot. (poor distribution habit) |
| DES-10 | 35 | 27.1 | 3319750 | In the artificial botanical garden, there is hardened ground around it. (poor distribution habit) |
| QCS-10 | 38.9 | 24.5 | 3707365 | 4 m from the road. (good distribution habit) |
| QCS-15 | 43.6 | 22.5 | 4277160 | Close to highways and drains. (poor distribution habit) |
| QCS-12 | 42.7 | 23.5 | 4284732 | On the platform, 4 m from the road. (good distribution habit) |
| PZS-2 | 42.3 | 30 | 5367870 | In a temple. (poor distribution habit) |
| SQ-1 | 50.3 | 28.3 | 7160155 | In the farmyard. (poor distribution habit) |
| TGS-3 | 66.9 | 22.5 | 10070120 | On the rocky cliff beside the highway. (poor distribution habit) |
| DMS-1 | 71.7 | 27.9 | 14343080 | In a temple. (poor distribution habit) |
